# Tankyrase inhibitors suppress hepatocellular carcinoma cell growth via modulating the Hippo cascade

**DOI:** 10.1371/journal.pone.0184068

**Published:** 2017-09-06

**Authors:** Jiaoyuan Jia, Yu Qiao, Maria G. Pilo, Antonio Cigliano, Xianqiong Liu, Zixuan Shao, Diego F. Calvisi, Xin Chen

**Affiliations:** 1 Department of Oncology and Hematology, The Second Hospital, Jilin University, Changchun, China; 2 Department of Bioengineering and Therapeutic Sciences and Liver Center, University of California, San Francisco, California, United States of America; 3 Department of Oncology, Beijing Hospital, National Center of Gerontology, Beijing, China; 4 Institue of Pathology, University Medicine Greifswald, Greifswald, Germany; 5 School of Pharmacy, Hubei University of Chinese Medicine, Wuhan, Hubei, China; 6 Lowell High School, San Francisco, California, United States of America; University of Navarra School of Medicine and Center for Applied Medical Research (CIMA), SPAIN

## Abstract

Previous data indicate that Tankyrase inhibitors exert anti-growth functions in many cancer cell lines due to their ability to inactivate the YAP protooncogene. In the present manuscript, we investigated the effect of Tankyrase inhibitors on the growth of hepatocellular carcinoma (HCC) cell lines and the molecular mechanisms involved. For this purpose, we performed cell proliferation assay by colony-forming ability in seven human HCC cells subjected to XAV-939 and G007-LK Tankyrase inhibitors. Noticeably, the two Tankyrase inhibitors suppressed the HCC cell growth in a dose-dependent manner. Furthermore, we found that Tankyrase inhibitors synergized with MEK and AKT inhibitors to suppress HCC cell proliferation. At the molecular level, Tankyrase inhibitors significantly decreased YAP protein levels, reduced the expression of YAP target genes, and inhibited YAP/TEAD luciferase reporter activity. In addition, Tankyrase inhibitors administration was accompanied by upregulation of Angiomotin-like 1 (AMOTL1) and Angiomotin-like 2 (AMOTL2) proteins, two major negative regulators of YAP. Altogether, the present data indicate that XAV-939 and G007-LK Tankyrase inhibitors could suppress proliferation of hepatocellular carcinoma cells and downregulate YAP/TAZ by stabilizing AMOTL1 and AMOTL2 proteins, thus representing new potential anticancer drugs against hepatocellular carcinoma.

## Introduction

Liver cancer is one of the most common malignant tumors and the second leading lethal cancer worldwide. Hepatocellular carcinoma (HCC) accounts for 90% of primary liver cancer with approximately 850,000 new cases annually [1]. In the early stage of the disease, resection, liver transplantation and local ablation have curative possibilities, whereas only chemoembolization and sorafenib administration provide limited survival benefits in advanced HCC [2]. Thus, there is an urgent need for novel therapies to improve significantly the outcome of HCC patients.

Tankyrases belong to a family of enzymes called poly ADP ribosyl polymerases (PARPs) and take part in various cellular and molecular functions, such as Wnt/β-catenin signaling, telomere homeostasis, glucose metabolism, and cell cycle progression. Tankyrases play an important role in a variety of diseases including cancer, inflammation, infections, and obesity [3]. Tankyrase1 and Tankyrase 2 are the two main members of the family and share 82% sequence identity. Of note, tankyrases have been found to be significantly elevated in human HCC [4]. In this tumor type, the XAV-939 and G007-LK selective Tankyrase1/2 inhibitors effectively block the Wnt–β-catenin signaling and induce growth restraint of HCC cells [4, 5].

Yes-associated protein (YAP) is a major core effector of the Hippo pathway, whose deregulation can promote cancer development in various tissues and organs [6]. Once activated, YAP acts as transcriptional co-activator by binding to TEA domain (TEAD) DNA binding proteins in the nucleus to initiate the expression of target genes [6]. In particular, recent studies have found that YAP exerts multiple functions on normal and cancer cells via its ability to interact with multiple signaling pathways, such as Wnt/β-catenin [7], Notch [8], EGF [9], and TGFβ [10] cascades, involved in cell proliferation, tissue regeneration, and stem cell self-renewal [6–11]. Angiostatin binding proteins, including Angiomotin-like 1 (AMOTL1) and Angiomotin-like 2 (AMOTL2), are critical negative regulators of YAP, as they inhibit its nuclear translocation [12]. Importantly, it has been found that Tankyrase proteins are able to interact with Angiomotins and enhance their degradation through the RNF146b E3 ligase, with consequent YAP activation [13–15].

Mounting evidence indicates that YAP is expressed in the vast majority of human HCC specimens [16–18], where its elevated levels and nuclear accumulation represent an independent prognostic indicator [16–18]. Due to the concomitant activation of YAP and Tankyrases observed in HCC, it is tempting to speculate that suppression of YAP activity via inhibition of Tankyrases might be therapeutically relevant for the treatment of this deadly disease. In the present study, to test this hypothesis, we examined the effects of two Tankyrase inhibitors, XAV-939 and G007-LK, on the *in vitro* growth of 7 human HCC cell lines. In particular, we investigated the effects of Tankyrase inhibitors on the YAP pathway. Furthermore, we evaluated whether Tankyrase inhibitors synergize with AKT or MEK inhibitors to inhibit HCC cell growth. The data obtained strongly suggest that Tankyrase inhibitors might be potentially effective anticancer drugs against HCC.

## Materials and methods

### Cell lines and culture

SNU-475, SNU-449, and SNU-398 HCC cells were obtained from ATCC (Manassas, VA); HLE and Huh7 cells from JCRB Cell Bank (Osaka, Japan); MHCC97H and MHCC97L cells from Liver Center Research Institute, Fudan University (Shanghai, China). HLE, Huh7, MHCC97-L, and MHCC97-H HCC cell lines were cultured in Dulbecco’s modified Eagle medium (DMEM) supplemented with 10% FBS, penicillin (100 U/mL), and streptomycin (100 μg/mL). SNU-475, SNU-449, and SNU-398 HCC cells were instead cultured in Roswell Park Memorial Institute 1640 (RPMI 1640) medium supplemented with 10% FBS, penicillin (100 U/mL), and streptomycin (100 μg/mL) in a humidified 5% CO_2_ incubator at 37°C. All cell lines underwent validation before being used in the experiments (Genetica DNA Laboratories, Burlington, NC)

### Clonogenic assay and assessment of proliferation and apoptosis

For clonogenic proliferation assay, the HCC cell lines were plated in triplicate at 1 X 10^3^/well in 6-well plates (SNU-449 0.5 X 10^3^/well). HCC cells were treated with 0.1% DMSO, or 2.5 μM, 5 μM, 10 μM, 20μM XAV-939 (ApexBio, Houston, TX) or G007-LK (ApexBio) until colonies became sufficiently large to be quantified. The medium with DMSO or inhibitors was replaced every 3 days. After 10–14 days, colonies were washed by PBS and dyed with crystal violet for at least 10 minutes at room temperature. Subsequently, the dye was washed off and colonies were counted. For cell proliferation or apoptosis assays, SNU-449 and HLE cells were grown in a 5% CO_2_ atmosphere, at 37°C, in RPMI Medium supplemented with 10% fetal bovine serum (FBS; Gibco, Grand Island, NY) and penicillin/streptomycin (Gibco). HCC cells were treated with 0.1% DMSO, or 2.5 μM, 5 μM, 10 μM, 20μM XAV-939 (ApexBio) or G007-LK (ApexBio), either alone or in combination with the MEK inhibitor U0126 (25 μM; Cell Signaling Technology Inc., Danvers, MA) or the AKT inhibitor MK-2206 (5 μM; ThermoFisher Scientific, Waltham, MA). Cell proliferation was analyzed using the BrdU Cell Proliferation Assay Kit (Cell Signaling Technology Inc.), while apoptosis was assessed with the Cell Death Detection Elisa Plus Kit (Roche Molecular Biochemicals, Indianapolis, IN), following the manufacturers’ protocols.

### Quantitative real-time reverse-transcription polymerase chain reaction

mRNA was isolated from fresh SNU-449 and HLE cells after 48h with 0.1% DMSO, 10μM XAV-939 or 10μM G007-LK treatment using the Quick-RNA^™^ MiniPrep (Zymo Research, Irvine, CA). Reverse transcription was conducted according to the manufacturer's instructions (Invitrogen Carlsbad, CA). The human primers used for PCR analysis were synthesized by Integrated DNA Technologies (Coralville, IA). The sequences of the primers were as follows: Connective tissue growth factor (CTGF), 5′-CCAATGACAACGCCTCCTG-3′ (forward) and 5′-GAGCTTTCTGGCTGCACCA-3′ (reverse); Cysteine rich angiogenic inducer 61 (Cyr61), 5′- GGTCAAAGTTACCGGGCAGT-3′ (forward) and 5′- GGAGGCATCGAATCCCAG-3′ (reverse); YAP, 5′-TAGCCCTGCGTAGCCAGTTA -3′ (forward) and 5′-TCATGCTTAGTCCACTGTCTGT -3′ (reverse); Glyceraldehyde-3-phosphate dehydrogenase (GAPDH), 5′-ATGGGGAAGGTGAAGGTCG-3′ (forward) and 5′-GGGGTCATTGATGGCAACAATA-3′ (reverse). Quantitative real-time polymerase chain reaction was performed with 100 ng of cDNA, using an ABI Prism 7000 Sequence Detection System and TaqMan Universal PCR Master Mix (ThermoFisher Scientific). Cycling conditions were: 10 min of denaturation at 95°C and 40 cycles at 95°C for 15 s and at 52°C for 1 min. Quantitative values were calculated by using the PE Biosystems Analysis software and expressed as N target (NT). NT = 2^-ΔCt^, wherein the ΔCt value of each sample was calculated by subtracting the average Ct value of the target gene from the average Ct value of the GAPDH gene.

### Dual-luciferase reporter assay

SNU-449 and HLE Cells were plated in triplicate in 24-well plate at 70–80% confluency and were co-transfected with 500ng of TEAD luciferase reporter plasmid (8xGTIIC-luciferase, Addgene plasmid #34615), 20ng of pRL-CMV Renilla luciferase plasmid (Promega, Madison, WI) and 1μL Lipofectamine 2000 per well (Invitrogen). The culture medium was replaced by Opti-MEM^®^ Reduced Serum. After 4-hour incubation, the transfection mixture was replaced by culture medium with 0.1%DMSO, 10μM XAV-939, or 10μM G007-LK. At 48 hours, the cells were rinsed in 1X PBS and harvested with Passive Lysis Buffer in a new tube on ice. Luciferase activity was measured using the Dual-Luciferase^®^ Reporter Assay System (Promega), according to the manufacturer’s protocol.

### Western blot analysis

Cells were washed in PBS and lysed in M-PER^™^ Mammalian Protein Extraction Reagent (ThermoFisher Scientific) containing the Halt^™^ Protease Inhibitor Cocktail (ThermoFisher Scientific). Nuclear extraction was performed using NE-PER^™^ Nuclear and Cytoplasmic Extraction Reagents (ThermoFisher Scientific). Protein concentration was quantified using the Pierce^™^ Microplate BCA Protein Assay Kit (ThermoFisher Scientific). Membranes were blocked in in 5% non-fat milk for 1 hour and incubated with the following primary antibodies: YAP (1:1000; Cell Signaling Technology), YAP/TAZ (1:1000; Cell Signaling Technology), AMOTL1 (1:1000; Sigma-Aldrich, St. Louis, MO), AMOTL2 (1:1000, Sigma-Aldrich), p-YAP(Ser127) (1:1000; Cell Signaling Technology), Histone H3 (1:2000; Cell Signaling Technology), β-tubulin (1:1000; abcam), t-AKT (1:1000; Cell Signaling Technology), p-AKT (1:1000; Cell Signaling Technology), t-ERK (1:1000; Cell Signaling Technology), and p-ERK (1:1000; Cell Signaling Technology). β-actin (1:5000; Sigma-Aldrich, St. Louis, MO) and GAPDH (1:10000; EMD Millipore, Billerica, MA) were used as a loading controls. Each primary antibody was followed by incubation with secondary antibody (1:5000; Jackson ImmunoResearch Laboratories Inc. West Grove, PA) for 1 hour and bands were revealed with the Super Signal West Dura (ThermoFisher Scientific).

### Statistical analysis

Statistical analysis was performed using Student’s t-test and Tukey-Kramer test. The data were expressed as the mean±SD of at least three independent experiments. P <0.05 was considered significant.

## Results

### XAV-939 and G007-LK restrain HCC cell growth *in vitro*

**Fig 1:**
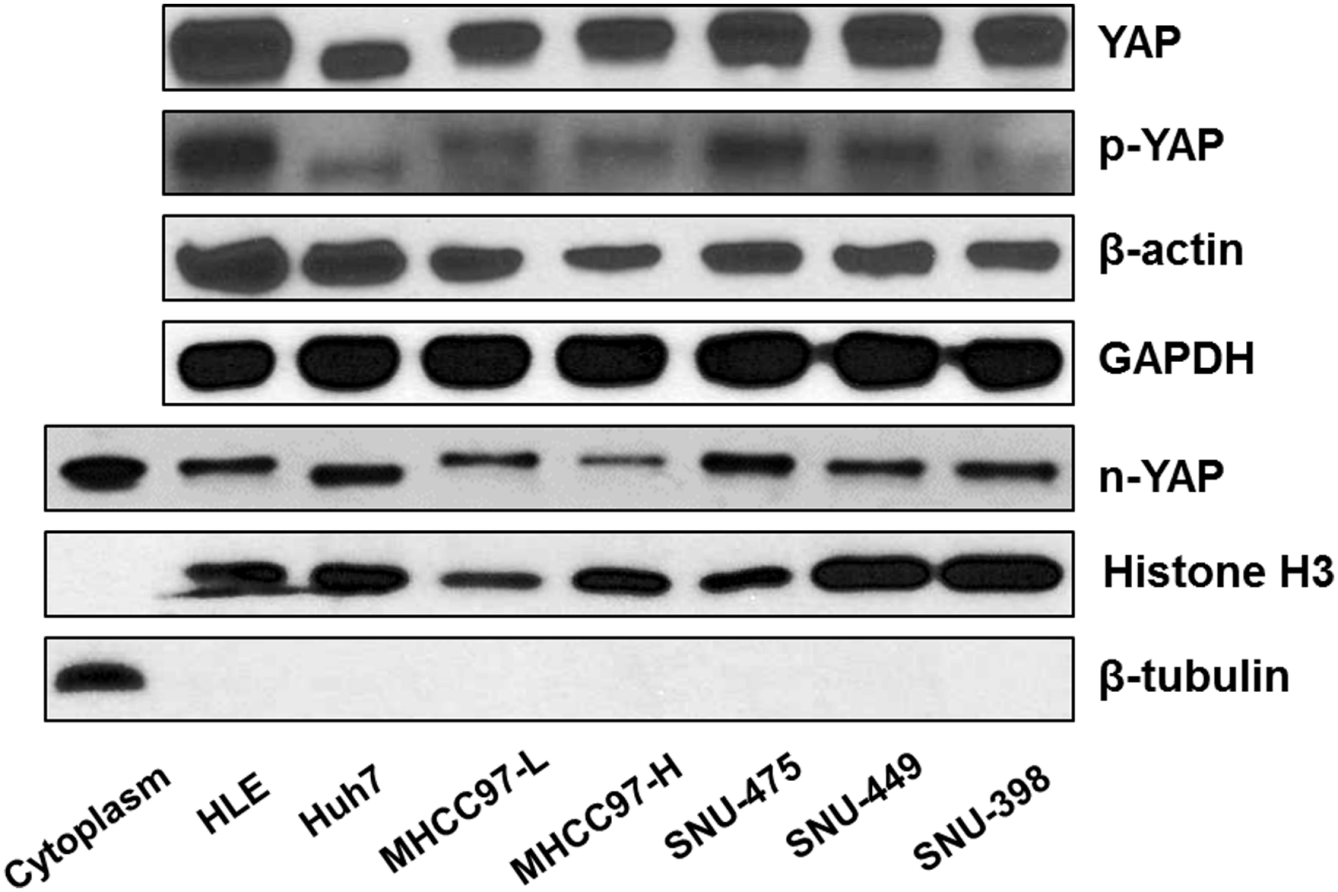
YAP expression in human HCC cell lines. Total YAP, phosphorylated (p-)YAP as well as nuclear YAP (n-YAP) protein levels were analyzed in a panel of 7 human HCC cell lines by Western blotting. β-actin and GAPDH were used as loading controls for total cell lysate. Histone H3 and β-tubulin were used as controls for nuclear and cytoplasmic extraction, respectively. The experiments were repeated twice.

**Fig 2:**
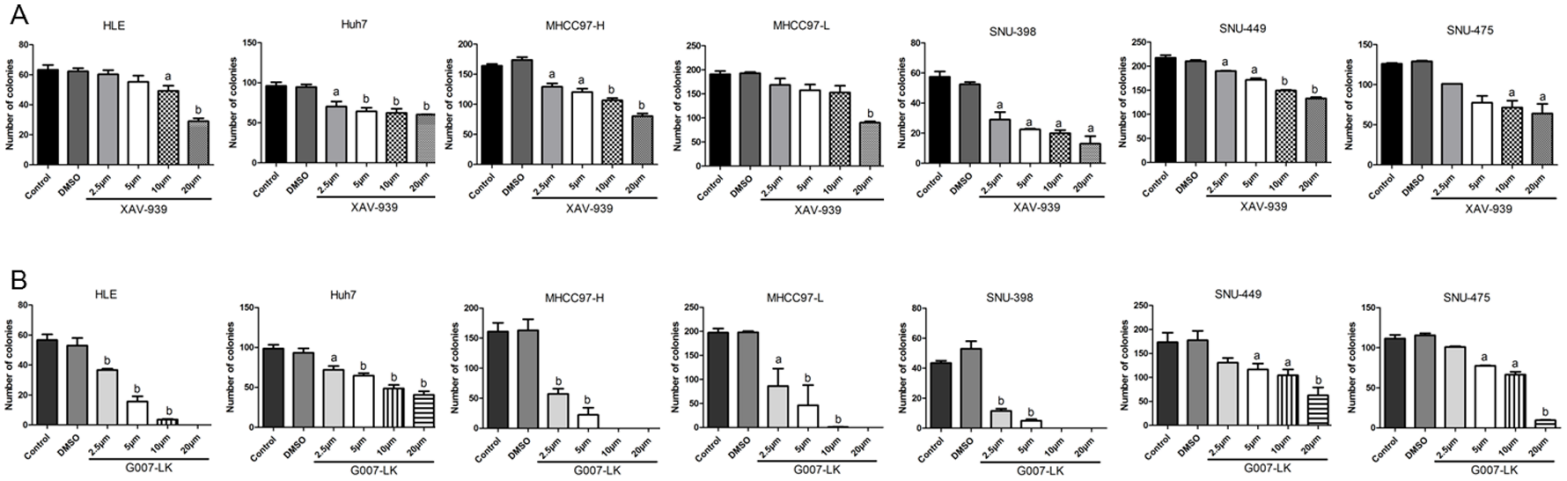
The XAV-939 and G007-LK Tankyrase inhibitors suppress HCC cell growth. Colony formation assay was used to determine the growth inhibitory effects of XAV-939 (A) and G007-LK (B) in the panel of 7 HCC cell lines. Student’s t-test: (a) p<0.05 vs DMSO; (b) p<0.01 vs DMSO. Colony formation assay was repeated three times for each drug in each cell line.

First, we determined YAP expression in a panel of 7 human HCC cell lines, including HLE, Huh7, MHCC97-L, MHCC97-H, SNU-475, SNU-449, and SNU-398 cells. We found that all 7 cells express YAP as well as phosphorylated YAP (p-YAP) (Fig 1 and S4 Fig). Importantly, analysis of nuclear protein extracts showed that YAP is localized in the nucleus of all HCC cell lines tested, thus indicating the ubiquitous activation of YAP in these cells (Fig 1 and S4 Fig). As Tankyrase inhibitors have been shown to suppress tumor cell growth via inhibiting YAP [13–15], we investigated the effect of two Tankyrase inhibitors, XAV-939 and G007-LK, in regulating HCC cell growth. Both XAV-939 and G007-LK displayed a dose-dependent growth inhibitory effect on all 7 HCC cell lines tested, as assessed by colony formation assay (Fig 2A and 2B). In general, G007-LK exhibited a higher growth inhibitory activity than that of XAV-939 (Fig 2A and 2B).

#### XAV-939 and G007-LK downregulate the levels of YAP and its targets by upregulating AMOTL1 and AMOTL2

**Fig 3:**
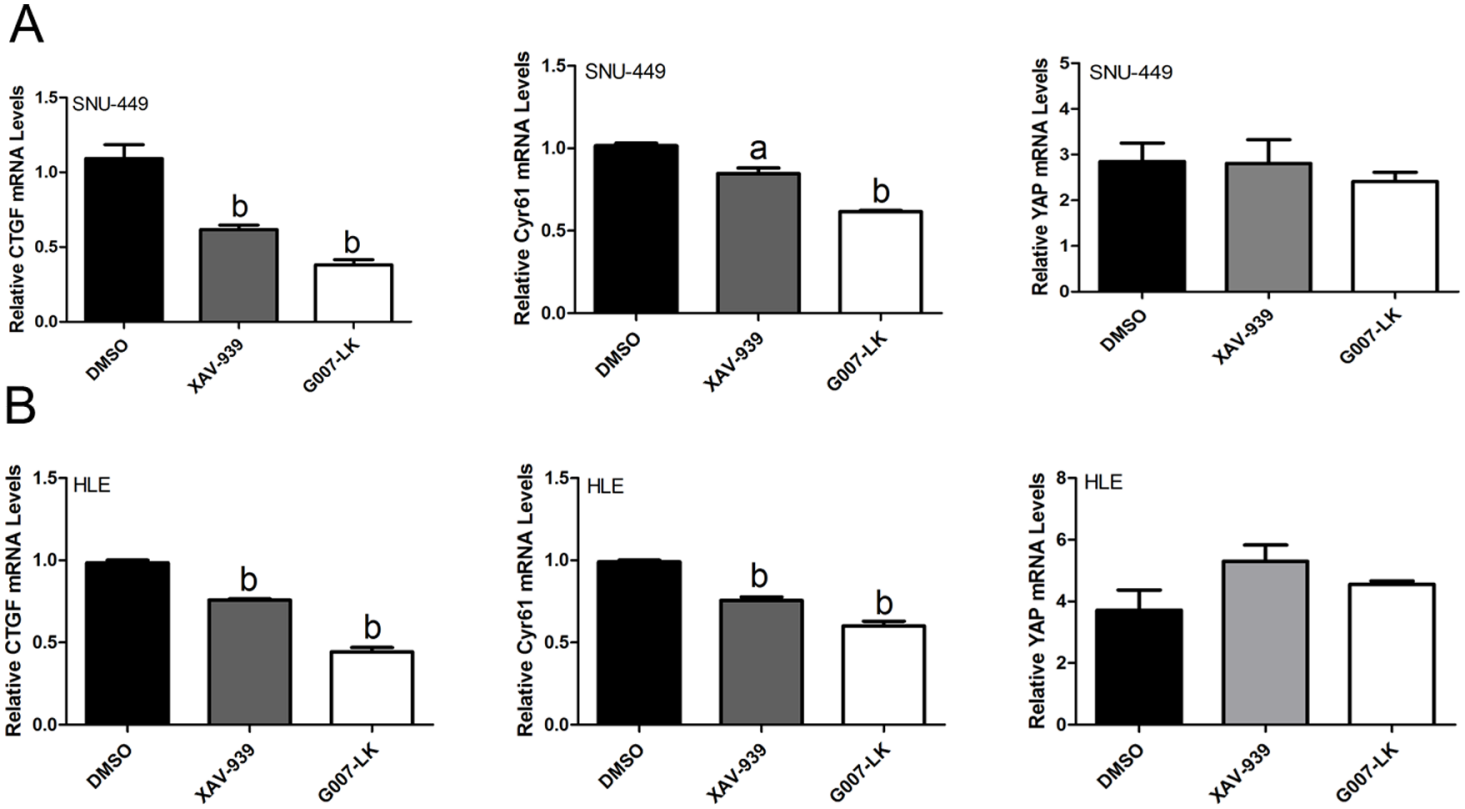
Tankyrase inhibitors decrease mRNA expression of YAP target genes in HCC cell lines. qRT-PCR analysis of mRNA expression of *CTGF*, *Cyr61*, and *YAP* in SNU-449 (A) and HLE (B) HCC cell lines upon treatment with XAV-939 and G007-LK inhibitors. Student’s t-test: (a) p<0.05 vs. DMSO; (b) p<0.01 vs. DMSO. qRT-PCR experiments were repeated three times.

**Fig 4:**
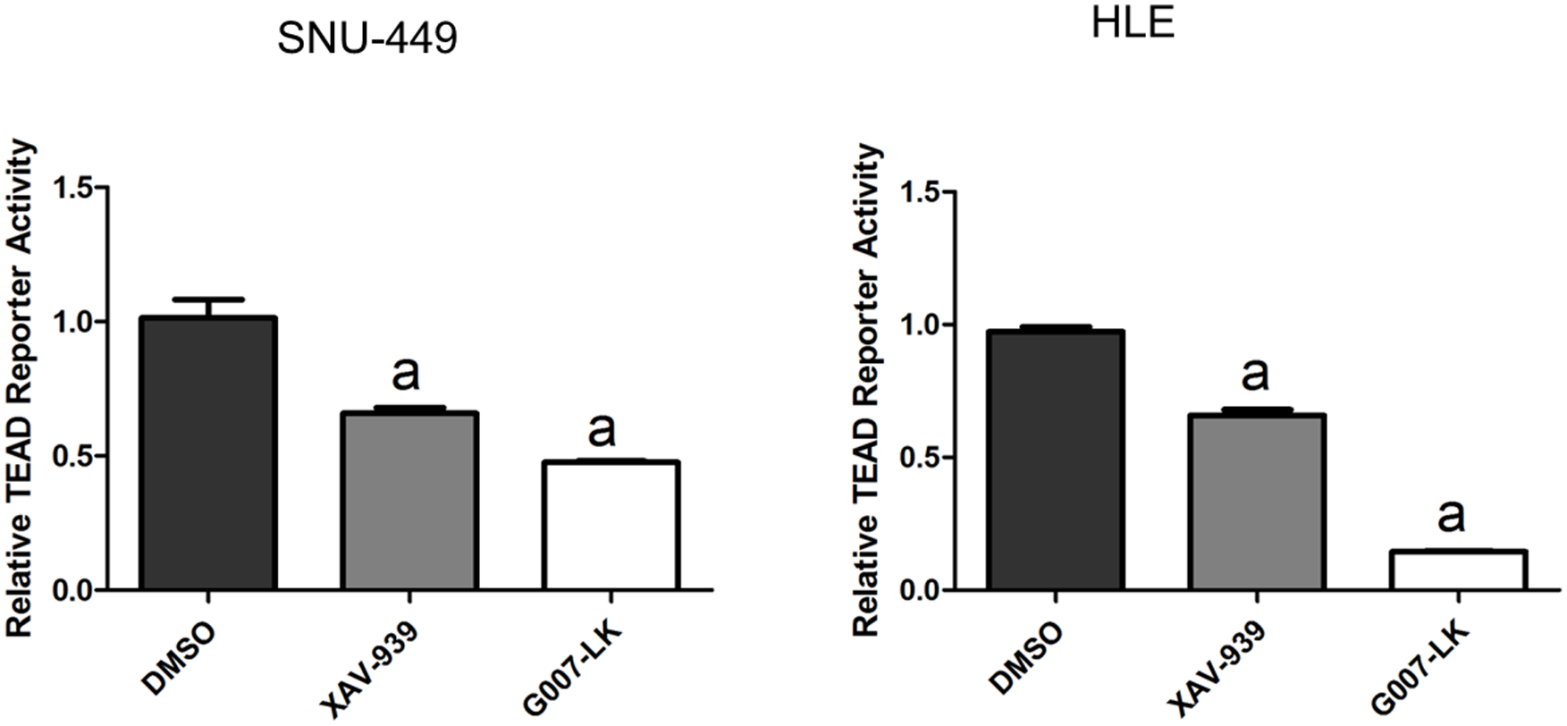
Tankyrase inhibitors suppress TEAD-luciferase activity in HCC cell lines. Relative TEAD reporter activity in SNU-449 and HLE HCC cells upon XAV-939 and G007-LK treatment is shown. Student’s t-test: (a) p<0.05 vs. DMSO; (b) p<0.01 vs. DMSO. Experiments were repeated three times.

**Fig 5:**
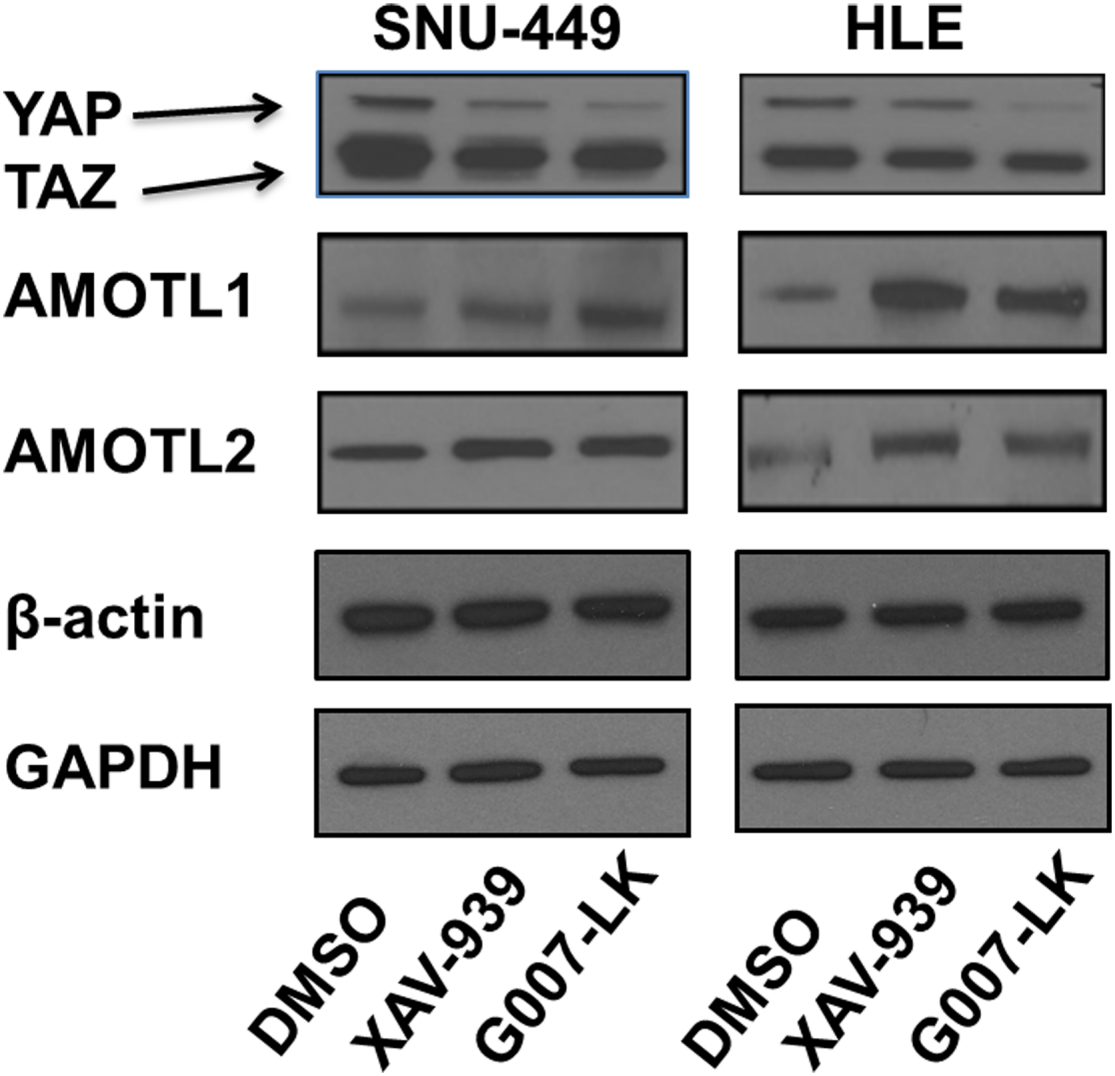
Tankyrase inhibitors reduce YAP protein levels and increase AMOTL1 and AMOTL2 protein expression in HCC cell lines. Western blot analysis of YAP, AMOTL1, and AMOTL2 in SNU-449 and HLE HCC cells upon XAV-939 or G007-LK treatment. β-actin and GAPDH were used as loading controls. Experiments were repeated twice.

Next, we investigated whether the XAV-939 and G007-LK Tankyrase inhibitors suppress YAP signaling in HCC cells. For this purpose, initially we assessed the mRNA expression of *YAP* and its downstream effectors *CTGF* and *Cyr61* by qRT-PCR. We found that the mRNA levels of CTGF and Cyr61, but not YAP, were significantly decreased in SNU-449 and HLE cells following the treatment with XAV-939 and G007-LK (Fig 3). In addition, YAP/TEAD luciferase reporter activity was significantly inhibited following the administration of XAV-939 and G007-LK (Fig 4), supporting the notion that Tankyrase inhibitors constrain the transcriptional activity of YAP. Consistent with the stronger growth inhibitory effects by G007-LK, this drug exhibited a more pronounced inhibitory activity towards YAP target gene expression and TEAD luciferase activity than that following XAV-939 administration (Figs 3 and 4). At the protein level, a decline in YAP amount and an increase in AMOTL1 and AMOTL2 expression were detected in cells treated XAV-939 or G007-LK when compared with control DMSO treated cells (Fig 5 and S5 Fig). Protein levels of TAZ, a paralog of YAP, were instead not affected by treatment with Tankyrase inhibitors (Fig 5 and S5 Fig).

In summary, our study demonstrates that Tankyrase inhibitors suppress YAP protein expression and activity via upregulation of AMOTL1 and AMOTL2 in HCC cells.

#### XAV-939 and G007-LK synergize with MEK and AKT inhibitors to suppress HCC cell proliferation *in vitro*

**Fig 6:**
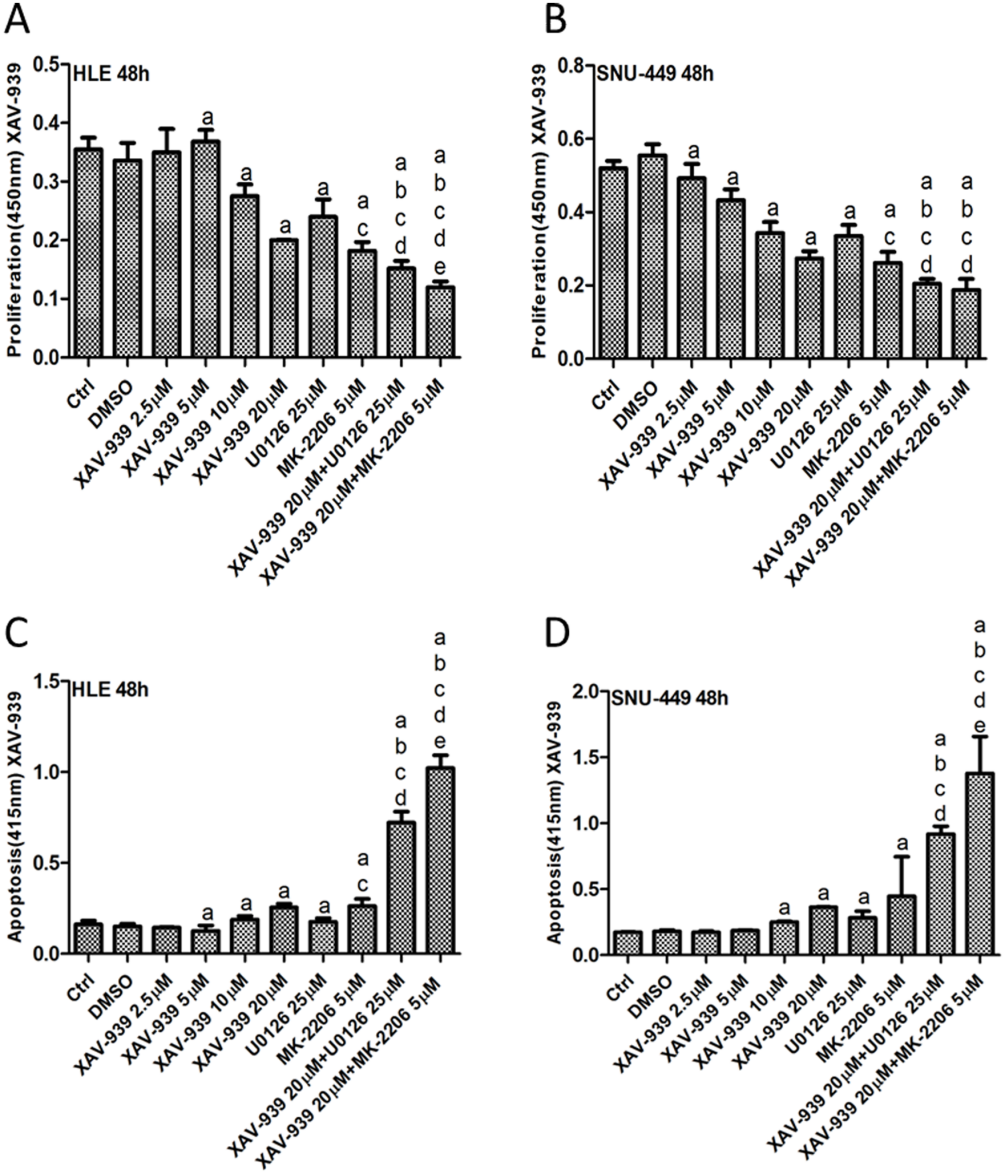
XAV-939 synergizes with U0126 and MK-2206 to inhibit HCC cell growth. Cell proliferation (A and B) and apoptosis (C and D) assays of HLE (A and C) and SNU-449 (B and D) cells treated with XAV-939, U0126 or MK-2206, either alone or in combination, for 48 hours. Tukey-Kramer test: p<0.05 (a) vs. DMSO; (b) vs. 20μM XAV-939 alone; (c) vs. 25μM U0126 alone; (d) vs. 5μM MK-2206 alone; (e) vs. XAV-939+U0126. Experiments were repeated three times in triplicate.

**Fig 7:**
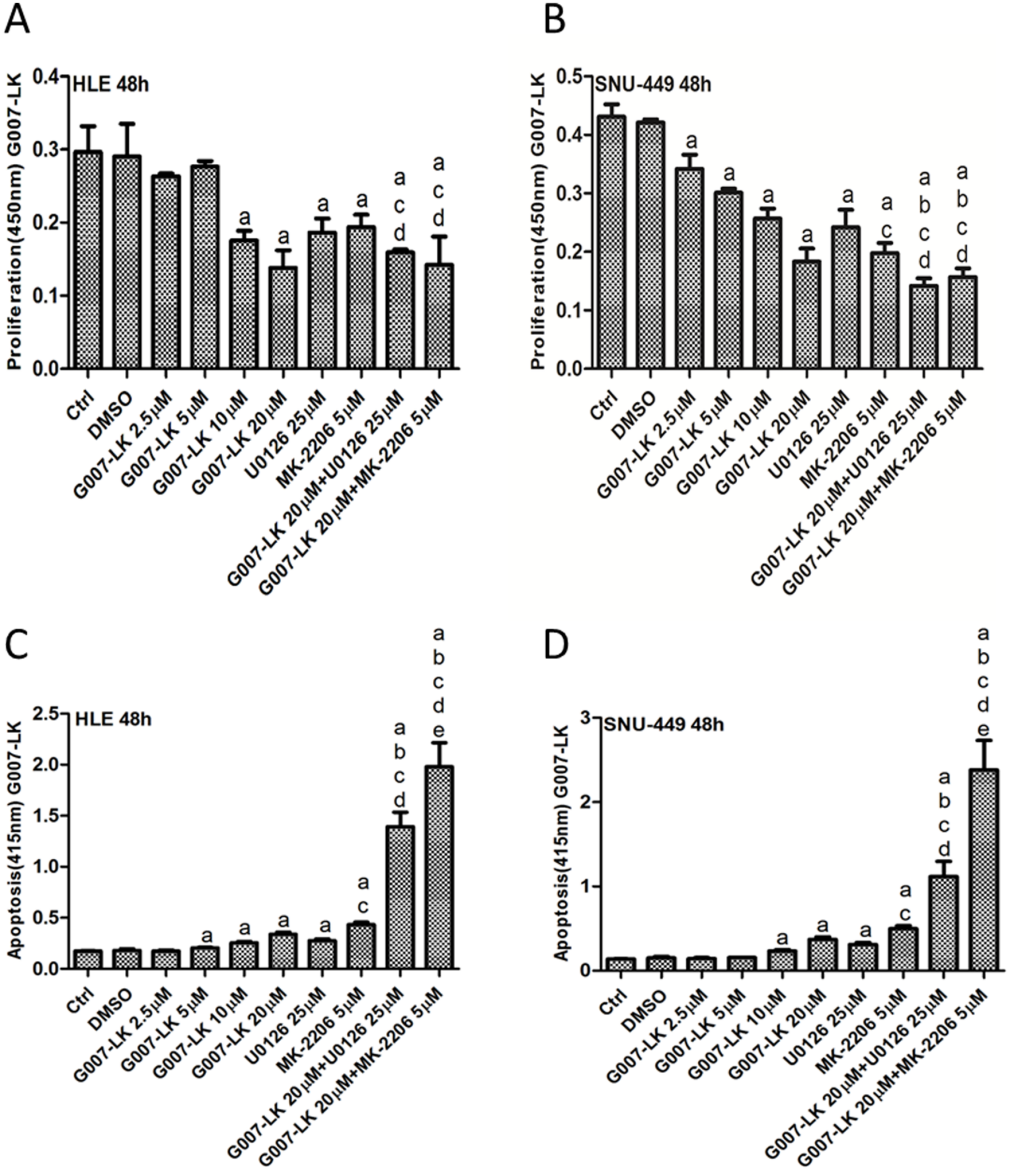
G007-LK synergizes with U0126 or MK-2206 to inhibit HCC cell growth. Cell proliferation (A and B) and apoptosis (C and D) assays of HLE (A and C) and SNU-449 (B and D) cells treated with G007-LK, U0126 or MK-2206, either alone or in combination for 48 hours. Tukey-Kramer test: p<0.05 (a) vs. DMSO; (b) vs. 20μM G007-LK alone; (c) vs. 25μM U0126 alone; (d) vs. 5μM MK-2206 alone; (e) vs. G007-LK+U0126. Experiments were repeated three times in triplicate.

As Ras/MAPK and AKT/mTOR signaling cascades are critical pathways for tumor cell growth, MEK and AKT inhibitors have been developed. In human HCC, frequent activation of Ras/MAPK and AKT/mTOR pathways has been previously detected [19–22]. Thus, it is not surprising that MEK and AKT inhibitors are effective in restraining the growth of HCC cells [23–25]. In the light of these previous findings, we hypothesized that combined inhibition of YAP and MEK or AKT pathways will lead to increased growth restraint of HCC cells. To test this hypothesis, we treated SNU-449 and HLE cell lines with XAV-939 or G007-LK Tankyrase inhibitors, either alone or in combination with MEK inhibitor U0126 or AKT inhibitor MK-2206. We found that treating cells with XAV-939, G007-LK, U0126 or MK-2206 alone led to decreased HCC cell proliferation and increased apoptosis. Importantly, XAV-939 or G007-LK synergized with U0126 and MK-2206 to further constrain HCC cell growth and promote massive apoptotic cell death (Figs 6 and 7, S1 and S2 Figs). At the biochemical level, XAV-939/G007-LK, U0126 or MK-2206 effectively inhibited YAP, phosphorylated/activated (p)-ERK and p-AKT levels, respectively, in SNU-449 and HLE cells (S3 and S6 Figs). The results suggest that combining Tankyrase inhibitors with MEK or AKT inhibitors might be a novel, effective therapeutic strategy for the treatment of HCC.

## Discussion

Deregulation of Hippo signaling pathway leading to the increased expression and activation of YAP has been found in multiple tumor types [6, 26], including HCC [16–18, 27, 28]. In this tumor type, inactivation of Hippo and high nuclear YAP expression has been associated with poor prognosis of the patients [16–18, 29, 30]. The pro-oncogenic role of YAP in hepatocarcinogenesis has been demonstrated both *in vitro* and *in vivo*. Indeed, overexpression of activated forms of YAP promotes HCC cell proliferation and migration [17], whereas silencing of YAP inhibits HCC cell growth and induces apoptosis [17, 31]. YAP has also been identified as a key survival factor mediating the resistance to chemotherapeutic drugs [32] in various tumor types, including HCC [33, 34]. Importantly, in animal studies, it was found that inhibiting YAP expression triggers regression of advanced stage HCC and drive hepatocyte differentiation [35]. Also, overexpression of an activated form of YAP together with a mutant form of phosphatidylinositol-4,5-bisphosphate 3-Kinase catalytic subunit alpha (PIK3CA) induced the development of HCC and cholangiocarcinomas in the mouse liver [36]. Taken together, this body of evidence strongly supports the development of anti-YAP based therapeutic strategies for the treatment of HCC.

Currently, drugs which can directly target the Hippo/YAP cascade are still very limited [6, 37]. Verteporfin, a photosensitizer that is clinically used for the treatment of macular degeneration, is the only small molecule that is able to directly target YAP mediated transcriptional activity [38]. In HCC, it has been found that Verteporfin is able to effectively inhibit HCC cell growth both *in vitro* [36, 38] and *in vivo* [38]. Verteporfin treatment also decreased liver preneoplastic foci formation in mice [18]. However, studies have also demonstrated that Verteporfin has multiple targets, and its *in vivo* efficacy is very limited. Therefore, Verteporfin is not an ideal YAP targeting drug [39]. An alternative approach for inhibiting YAP is to use YAP RNAi-lipid nanoparticles (siYAP-LNP). For instance, Fitamant J. *et al*. showed that siYAP-LNP can efficiently induce HCC regression in liver specific *Mst1/Mst2* knockout mice [35]. siYAP-LNP can also reverse liver enlargement in mice induced by Hippo inactivation [40]. However, the delivery efficacy of RNAi-lipid nanoparticles to human HCC and their effectiveness in the treatment of liver cancer remain to be determined. Clearly, it is imperative to identify additional approaches to successfully target YAP in the treatment of HCC.

Tankyrases are poly(ADP-ribose) polymerases that can induce the poly-ADP ribosylation of their substrates, leading to the change of subcellular localization or degradation of these substrates. Tankyrase substrates include a growing number of tumor suppressors, whose inactivation is implicated in cancer development and progression [13–15]. Therefore, Tankyrases have become attractive drug targets, and numerous small molecules able to inhibit Tankyrase activity have been developed, including G007-LK, NVP-TNKS656, WIKI4, and XAV-939 [41, 42]. *In vitro* and *in vivo* studies have demonstrated the excellent efficacy of these Tankyrase inhibitors for cancer treatment [43, 44]. Mechanistically, studies of Tankyrase inhibitors have been largely focusing on their ability to target and reactivate Axin proteins, thus leading to the inactivation of the Wnt/β-catenin signaling cascade [45, 46]. Recently, several studies suggest that Angiostatin binding protein family members (AMOT), which are involved in the negative regulation of YAP activity, are substrates of Tankyrases [13–15]. Tankyrase inhibitors, therefore, may inhibit YAP pathway by targeting AMOT proteins. However, whether Tankyrase inhibitors target YAP in HCC has not been investigated before. In the present study, we tested the hypothesis that Tankyrase inhibitors suppresses HCC cell growth via targeting the YAP pathway. For this purpose, we have chosen a panel of 7 HCC cell lines with high expression of YAP. Our findings indicate that the Tankyrase inhibitors G007-LK and XAV-939 are effective in inducing growth restraint in these cells. In addition, we show that G007-LK and XAV-939 cooperate with MEK and AKT inhibitors to further suppress HCC cell growth. Mechanistically, we demonstrated that, in HCC cells, administration of Tankyrase inhibitors increases AMOTL1 and AMOTL2 protein expression, leading to decreased YAP protein levels and transcriptional activity. Tankyrase treatment did not result in consistent changes of Ras/MAPK and AKT/mTOR pathways, two critical signaling cascades regulating HCC cell growth and survival (S3 Fig). This finding was expected as Tankyrases do not directly target these pathways. Importantly, the concomitant treatment of HCC cells with Tankyrase inhibitors and MEK or AKT inhibitors led to increased cell growth suppression (Figs 6 and 7). The precise mechanism(s) underlying this synergistic anti-growth effect remains to be defined. Nonetheless, our results strongly support further investigation on these combined therapies for HCC treatment.

It is interesting to note that activation of YAP and Wnt/β-catenin pathways characterizes two distinct molecular classes of human HCC. Recent studies have shown that co-activation of YAP and β-catenin frequently occurs in hepatoblastoma, a pediatric form of liver tumor, but rarely in HCC [47]. HCCs with activated β-catenin tend to be chromosomal stable [48], whereas YAP activation has recently been linked to chromosomal instability in HCCs [49]. Most importantly, inhibition of YAP in mouse liver tumors induced Wnt/β-catenin pathway activation and hepatocyte differentiation [35]. As Tankyrase inhibitors can target both YAP and Wnt/β-catenin cascades, these drugs may have rather broad anti-HCC efficacy. Furthermore, in HCCs with high YAP activity, Tankyrase inhibitors may prevent Wnt activation induced by YAP suppression.

Altogether, our study supports the hypothesis that Tankyrase inhibitors may represent novel and effective small molecules targeting YAP for HCC treatment. Obviously, additional experiments *in vivo* are required to evaluate the effectiveness of Tankyrase inhibitors for HCC treatment. For this purpose, appropriate preclinical models such as genetically engineered mouse (GEM) models and patient derived xenograft (PDX) models of liver cancer should be subjected to administration of Tankyrase inhibitors. In addition, it would be crucial to identify biomarkers, such as YAP nuclear localization or AMOTL1/2 downregulation, which can predict Tankyrase inhibitors’ therapeutic efficacy against HCC. Finally, the combined treatment of Tankyrase inhibitors with other drugs, including novel targeted therapy drugs or immunotherapy drugs, should be explored in detail *in vitro* and *in vivo* for HCC treatment.

## Supporting information

S1 Fig XAV-939 synergizes with U0126 or MK-2206 to inhibit HCC cell growth. Cell proliferation (A and B) and apoptosis (C and D) assays of HLE (A and C) and SNU-449 (B and D) cells treated with XAV-939, U0126 or MK-2206, either alone or in combination for 72 hours. Tukey-Kramer test: p<0.05 (a) vs. DMSO; (b) vs. 20μM XAV-939 alone; (c) vs. 25μM U0126 alone; (d) vs. 5μM MK-2206 alone; (e) vs. XAV-939+U0126. Experiments were repeated three times in triplicate.
(TIF)

S2 Fig G007-LK synergizes with U0126 or MK-2206 to inhibit HCC cell growth. Cell proliferation (A and B) and apoptosis (C and D) assays of HLE (A and C) and SNU-449 (B and D) cells treated with G007-LK, U0126 or MK-2206, either alone or in combination for 72 hours. Tukey-Kramer test: p<0.05 (a) vs. DMSO; (b) vs. 20μM G007-LK alone; (c) vs. 25μM U0126 alone; (d) vs. 5μM MK-2206 alone; (e) vs. G007-LK+U0126. Experiments were repeated three times in triplicate.
(TIF)

S3 Fig Regulation of YAP, p-ERK and p-AKT expression by tankyrase inhibitors, MEK inhibitor, or AKT inhibitor. Western blot analysis of YAP, phosphorylated (p)-AKT and p-ERK levels in SNU-449 and HLE HCC cells upon treatment with XAV-939 and G007-LK, either alone or in combination with U0126 or MK-2206. Total (t-) AKT, ERK, and GAPDH were used as loading controls. Experiments were repeated twice.
(TIF)

S4 Fig Scanned images for Western blotting for Fig 1. (TIF)

S5 Fig Scanned images for Western blotting for Fig 5. (TIF)

S6 Fig Scanned images for Western blotting for S3 Fig. (TIF)
