# Supplementary figures and images for: Tankyrase inhibitors suppress hepatocellular carcinoma cell growth via modulating the Hippo cascade
Source: PLoS One. 2017 Sep 6;12(9):e0184068. doi: 10.1371/journal.pone.0184068 (PMC5587291; doi:10.1371/journal.pone.0184068)

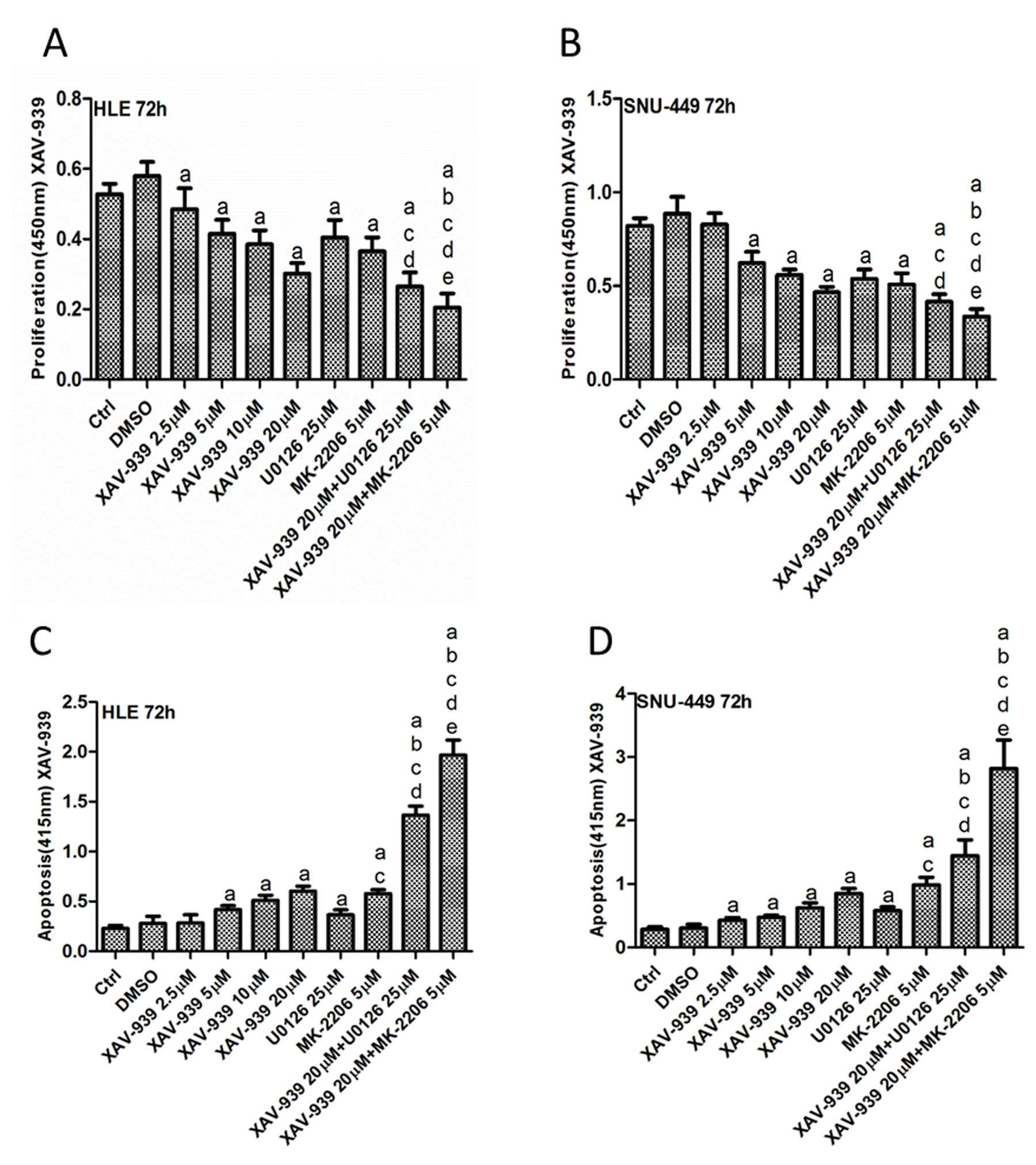

Supplement: S1 Fig — Cell proliferation (A and B) and apoptosis (C and D) assays of HLE (A and C) and SNU-449 (B and D) cells treated with XAV-939, U0126 or MK-2206, either alone or in combination for 72 hours. Tukey-Kramer test: p<0.05 (a) vs. DMSO; (b) vs. 20μM XAV-939 alone; (c) vs. 25μM U0126 alone; (d) vs. 5μM MK-2206 alone; (e) vs. XAV-939+U0126. Experiments were repeated three times in triplicate. (TIF) [file pone.0184068.s001.tif]

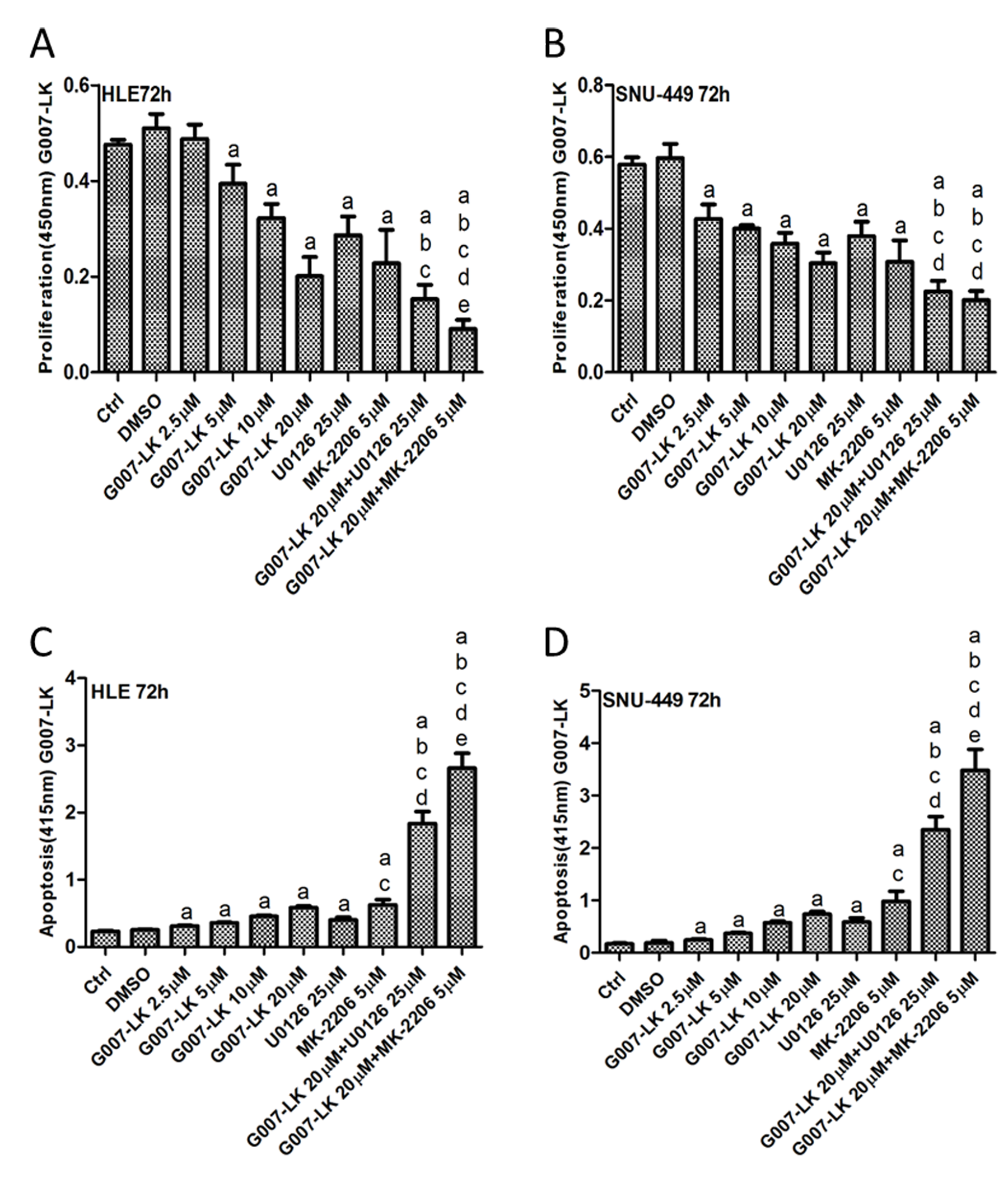

Supplement: S2 Fig — Cell proliferation (A and B) and apoptosis (C and D) assays of HLE (A and C) and SNU-449 (B and D) cells treated with G007-LK, U0126 or MK-2206, either alone or in combination for 72 hours. Tukey-Kramer test: p<0.05 (a) vs. DMSO; (b) vs. 20μM G007-LK alone; (c) vs. 25μM U0126 alone; (d) vs. 5μM MK-2206 alone; (e) vs. G007-LK+U0126. Experiments were repeated three times in triplicate. (TIF) [file pone.0184068.s002.tif]

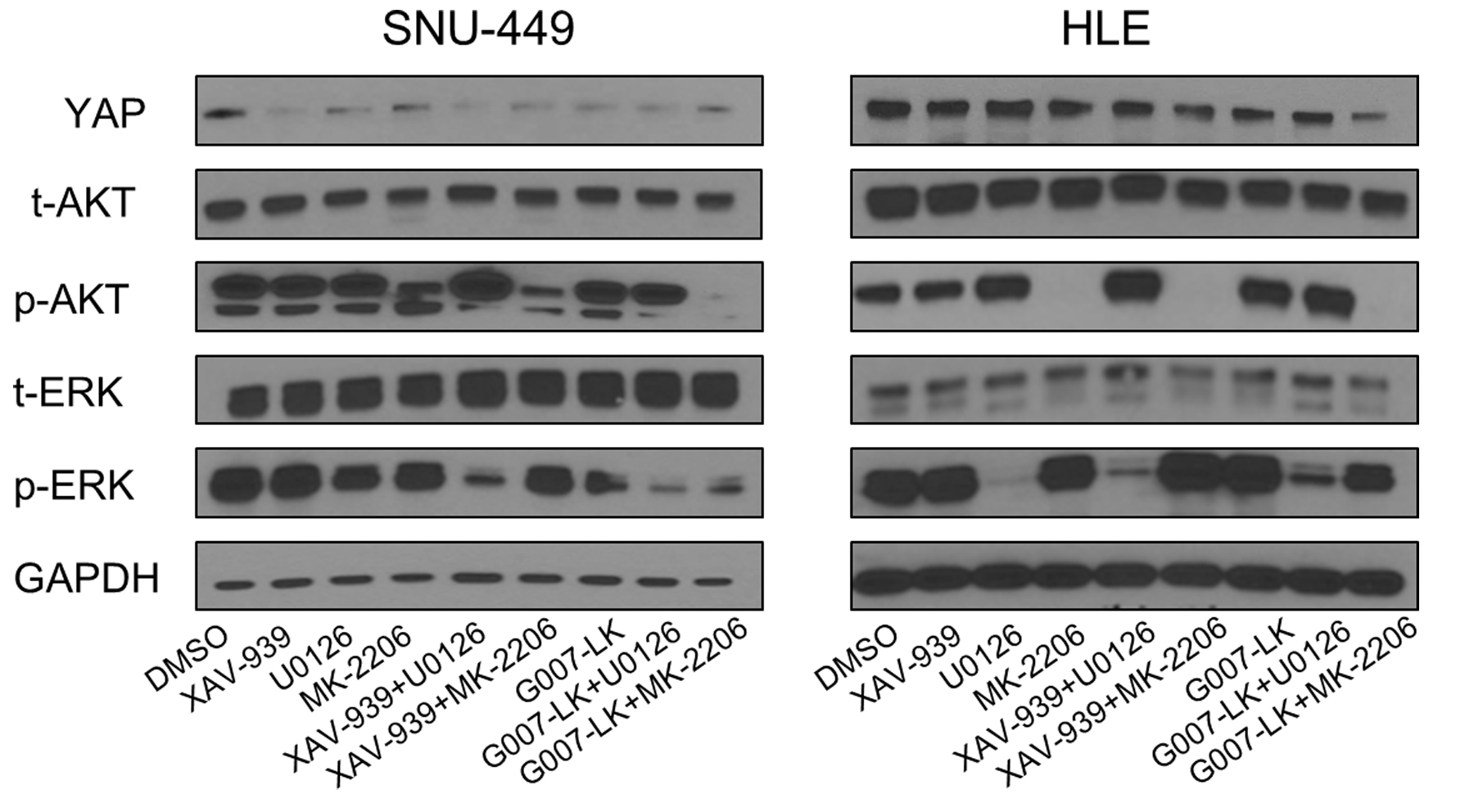

Supplement: S3 Fig — Western blot analysis of YAP, phosphorylated (p)-AKT and p-ERK levels in SNU-449 and HLE HCC cells upon treatment with XAV-939 and G007-LK, either alone or in combination with U0126 or MK-2206. Total (t-) AKT, ERK, and GAPDH were used as loading controls. Experiments were repeated twice. (TIF) [file pone.0184068.s003.tif]

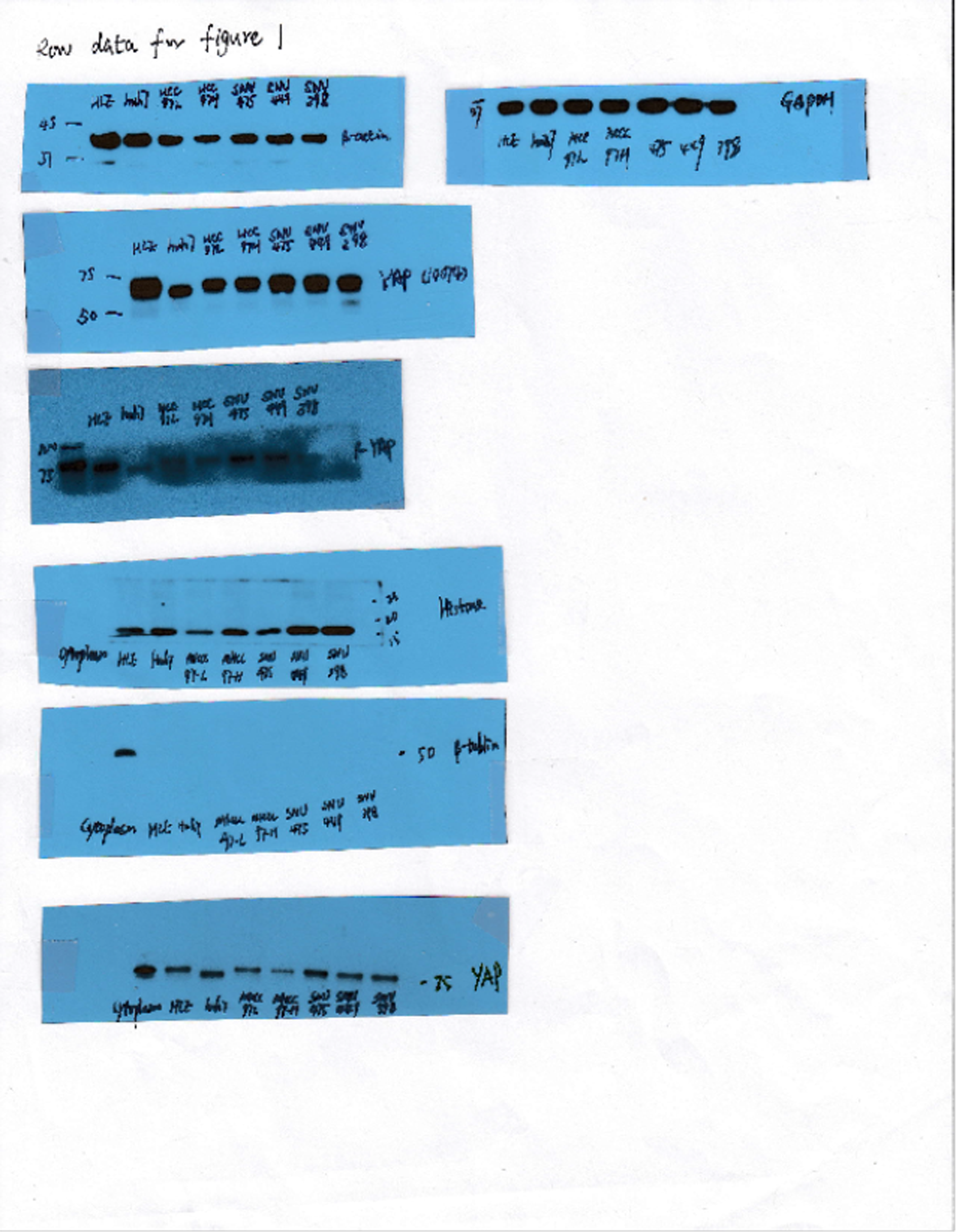

Supplement: S4 Fig — (TIF) [file pone.0184068.s004.tif]

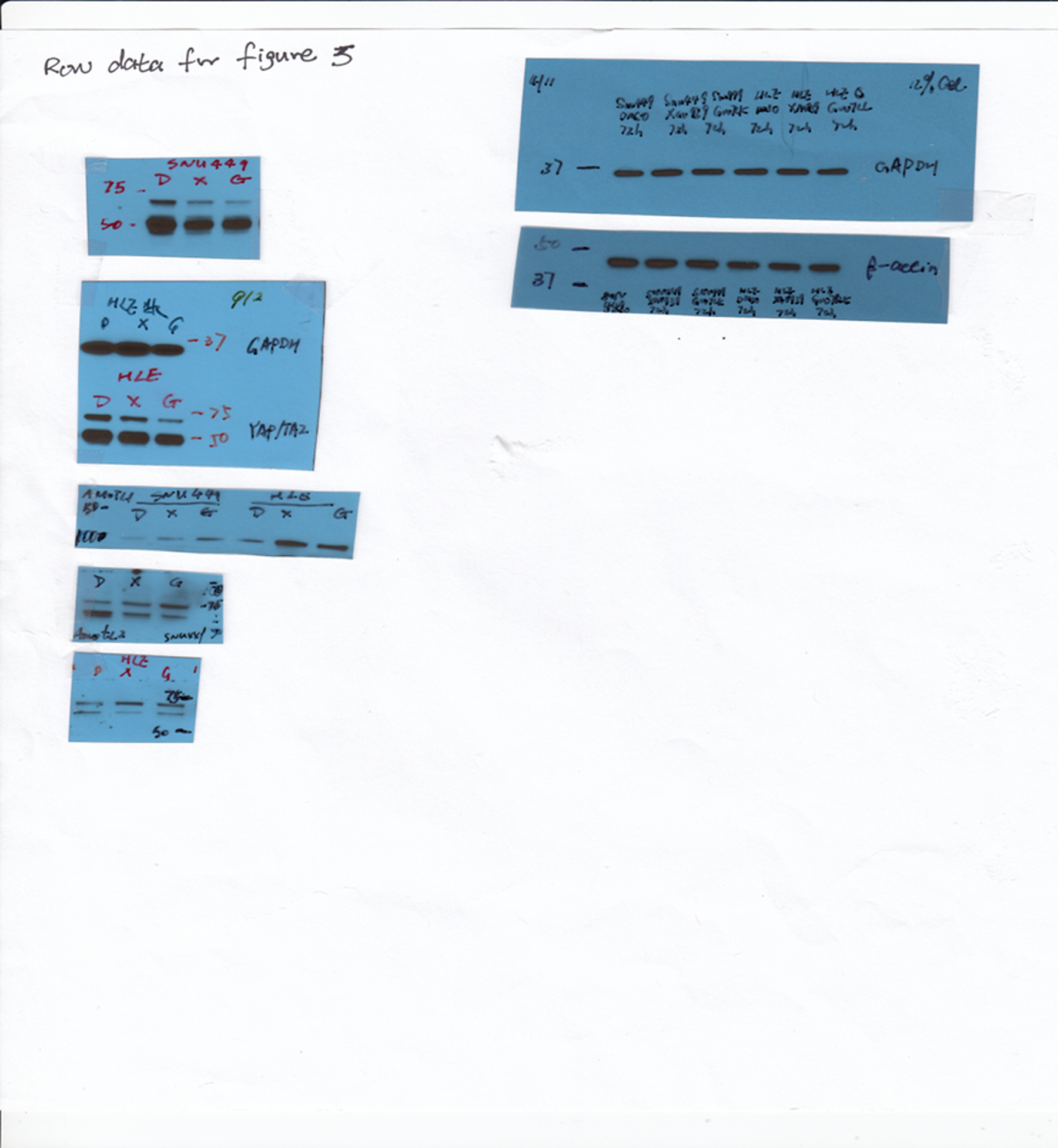

Supplement: S5 Fig — (TIF) [file pone.0184068.s005.tif]

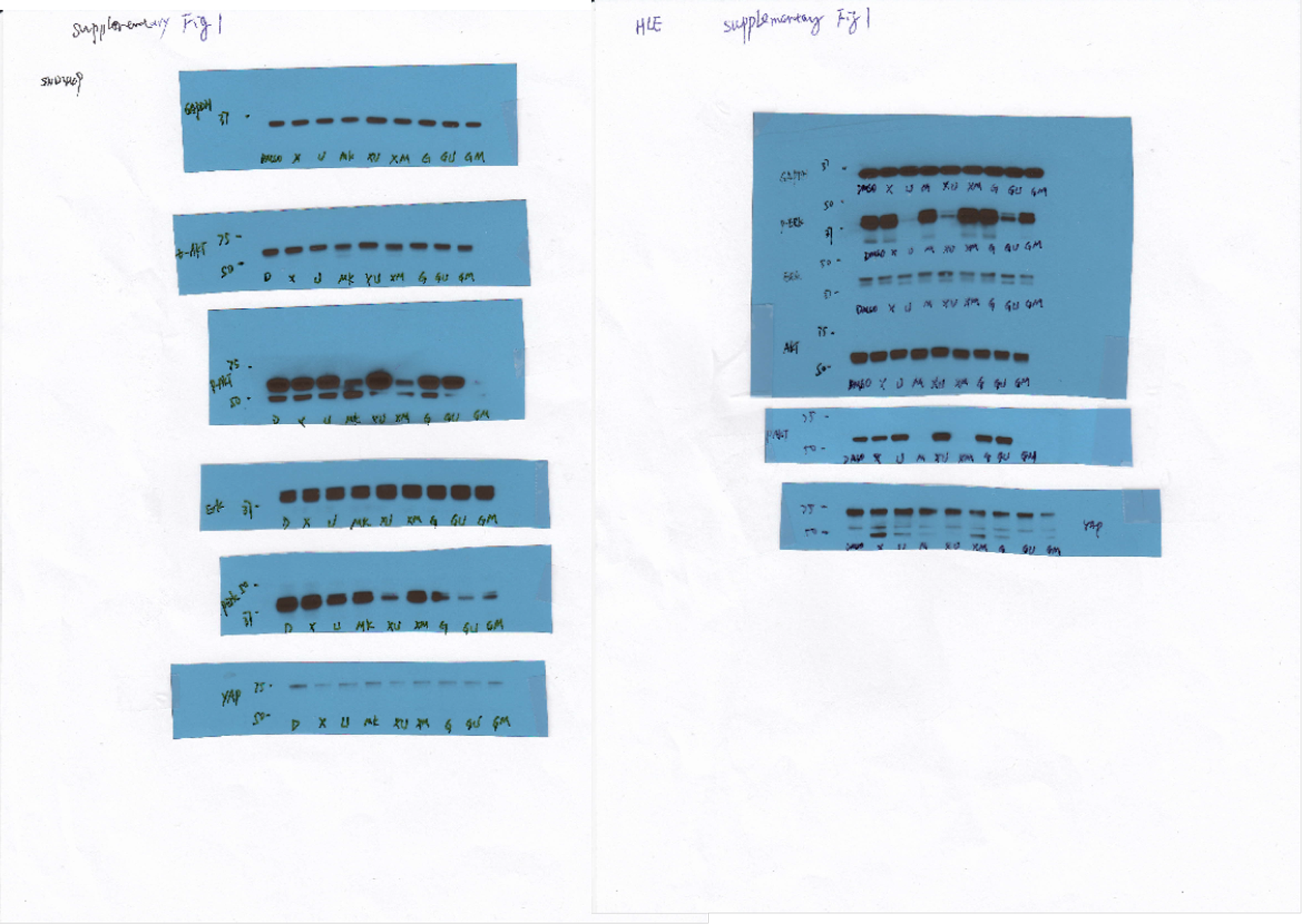

Supplement: S6 Fig — (TIF) [file pone.0184068.s006.tif]
